# Follow-up on commitments at the Third Global Forum on Human Resources for Health: Indonesia, Sudan, Tanzania: “A commitment is a promise, a promise is a debt”

**DOI:** 10.1186/s12960-016-0112-0

**Published:** 2016-04-26

**Authors:** Gilles Dussault, Elsheikh Badr, Hartiah Haroen, Martin Mapunda, Achmad Soebagja Tancarino Mars, Kirana Pritasari, Giorgio Cometto

**Affiliations:** Global Health and Tropical Medicine, WHO Collaborating Center on Health Workforce Policy and Planning, Instituto de Higiene e Medicina Tropical, Lisbon, Portugal; Sudan Medical Specialization Board, Khartoum, Sudan; WHO-Indonesia country office, Jakarta, Indonesia; Ministry of Health and Social Welfare, Dar Es Salaam, Tanzania; Ministry of Health, Jakarta, Indonesia; Global Health Workforce Alliance, World Health Organization, Geneva, Switzerland

**Keywords:** Health workforce commitments, Health workforce policies, Policy analysis, Indonesia, Sudan, Tanzania, Third Global Forum, Monitoring, Evaluation

## Abstract

This study sought to assess actions which Indonesia, Sudan, and Tanzania took to implement the health workforce commitments they made at the Third Global Forum on Human Resources for Health (HRH) in November 2013. The study was conducted through a survey of published and gray literature in English and field research consisting of direct contacts with relevant ministries and agencies. Results show that the three countries implemented interventions to translate their commitments into actions. The three countries focused their commitments on improving the availability, geographical accessibility, quality of education, and performance of health workers. The implementation of the Recife commitments primarily entailed initiatives at the central level, such as the adoption of new legislation or the development of accreditation mechanisms. This study shows that action is more likely to take place when policy documents explicitly recognize and document HRH problems, when stakeholders are involved in the formulation and the implementation of policy changes, and when external support is available. The Recife Forum appears to have created an opportunity to advance the HRH policy agenda, and advocates of health workforce development in these three countries took advantage of it.

## Background

A discussion paper presented at the Third Global Forum on Human Resources for Health^1^ (HRH) in November 2013 concluded that there was *No Health without a Workforce* [1]. The paper acknowledged that progress had been made in addressing the “health workforce crisis” described in the *World Health Report 2006: Working Together for Health* [2] but concluded that much more effort was needed to develop a “skilled, well-trained and motivated workforce” in order to achieve universal health coverage (UHC), a goal set by the United Nations General Assembly in December 2012 [3]. The organizers of the Recife Forum, the Global Health Workforce Alliance, the World Health Organization (GHWA-WHO), the Pan-American Health Organization, and the Government of Brazil, challenged participating countries and organizations to make specific commitments to improve the availability, accessibility, acceptability, and quality of their health workforce; representatives of 57 countries and 27 organizations did so. Commitments covered areas such as education, employment, management, deployment, motivation, and retention of health workers.^2^

The objective of this paper is to assess progress of the early stages of implementation of the Recife commitments and to identify barriers and facilitators to such progress. Indonesia (Southeast Asia Region), Sudan (Eastern Mediterranean Region), and Tanzania (Africa Region) were selected from the range of countries that made commitments. We first present background information on the three countries and then the criteria for their selection, the information sources, and the strategy of analysis of the evidence collected. The findings are then presented for each country, followed by broader lessons learned.

Table 1 presents basic health, workforce, and expenditure indicators on the three countries for the most recent year available, which is usually 2013. Table 2 presents contextual information on the health workforce situation in the three countries.

**Table 1:** Basic health and care system statistics 2013: Indonesia, Sudan, and Tanzania

Indicator\country	Indonesia	Sudan	Tanzania
Population (000)	249 866	37 964	49 253
Gross national income per capita (USD)^a^	3 580	1 550	630
Life expectancy at birth (men)	69	61	61
Life expectancy at birth (women)	73	65	65
Maternal mortality (per 100 000 live births)	190	360	410
Under-5 mortality (per 1 000 live births)	29	77	52
Infant mortality (per 1 000 live births)	25	51	36
N. of physicians per 10 000 (2007–2013)	2.0	2.8	0.3
N. of nurses and midwives per 10 000 (2007–2013)	13.8	8.4	4.4
Total expenditure on health (US$ PPP) as % of GDP (2012)	3.0	6.7	7.1
Public expenditure as % of total health expenditure (2012)	39.6	22.5	39.0
Expenditure on health as % of total Government expenditure (2012)	6.6	11.1	11.2

^a^World Bank indicators: http://data.worldbank.org/indicator/NY.GNP.PCAP.CD

Source: WHO, *World Health Statistics 2015*, World Health Organization, Geneva; http://www.who.int/gho/publications/world_health_statistics/2015/en/

**Table 2:** The health workforce policy context in Indonesia, Sudan, and Tanzania

Indonesia	Sudan	Tanzania
*Availability*	*Availability*	*Availability*
The production of qualified health workers has increased significantly in the last 10 years. The number of medical schools went from 40 in 2003 to 72 (of which 43 were private) in 2013; there were 33 736 physicians in 2010 and 81 131 in 2014, an increase of 140% [19]. There are 313 diploma, 275 bachelor, and 9 master nursing programs formally recognized.^b^ There were 169 697 nurses working in health facilities in 2010 and 295 508 in 2014, an increase of 74%. *Accessibility* The imbalanced distribution and the insufficient quality of the health workforce are major challenges [20] and an obstacle to achieving universal health coverage [21]. Among the 9 550 health centers, 9.8% are without doctors, 23% are without nutritionists, and 61.7% have no health promotion workers [19]. The geographical distribution of nurses and midwives is less uneven than that of doctors, but there are still important variations [19, 22]. The specificities of the numerous islands and of rural areas outside the main island of Java pose additional challenges to health workforce policies [23–25]. *Policy and regulation* The current government’s three priorities for HRH are as follows: *production*, *distribution*, and improving the *quality and performance* of health workers by ensuring that education and training institutions meet national standards [4]. In 2014, one third of medical undergraduate programs were not accredited, and the situation was similar in other health professions. A national examination was introduced in 2013 for medical, nursing, and midwifery students as a condition of access to the register; a similar exam is planned for pharmacy and dentistry graduates [23].	The availability of physicians, nurses and midwives is low in spite of the rapid growth of medical schools from 4 in 1990 to 28 in 2006, and to 34 in 2012, producing about 3 000 doctors per year; the number of nursing and midwifery schools rose from 18 in 2006 to 55 in 2013. *Accessibility* There are major variations in the geographical distribution of health workers: 65% of specialist physicians and 58% of technicians are in the capital, where about 20% of the population lives. Emigration of health workers is a major challenge for the country, particularly among physicians. Not only new graduates but also experienced physicians have left the country to work in Saudi Arabia, the USA, and the UK—though numbers registered there have diminished in recent years because of restrictions on hiring health personnel from poor countries—and also Ireland^c^ [26–28]. The public sector employs 62% of all health workers, the private sector 34%, and the military, university, police, and voluntary sectors 1% each. It is estimated that 90% of health professionals work in both the public and the private sector [26]. *Policy and regulation* The Ministry of Higher Education is responsible for pre-service training. The Sudan Medical Council registers doctors, pharmacists, and dentists, and the National Council for Medical and Health Professions regulates the rest of the qualified health workforce. There is a *National Human Resources for Health Strategic Plan 2012–2016* which identified the main challenges as “developing capacity for HRH planning and policies, augmenting equitable distribution, improving performance management systems, improving health workforce production, education and training and strengthening HRH functions at decentralized levels” [29, 30]. There has been a HRH Observatory since 2007 (http://www.who.int/workforcealliance/members_partners/member_list/nhrhobs_sudan/en/) and a Council for Coordination, composed of representatives of ministries, training institutions, the medical council, trade unions, aid agencies and the private sector, meets quarterly to discuss HRH issues [27].	The Ministry of Health and Social Welfare (MoHSW) recognized that shortage of personnel and imbalances in the geographical distribution and in the skill mix of health workers are a major impediment to achieving the health MDGs [31, 32]. In March 2013, there were 64 449 health workers of all categories, including 12 074 in the private sector,^d^ represented 36.4% of the requirement according to MoHSW standards; qualified workers included 1 135 medical doctors, 1 741 assistant medical officers, 5 950 clinical officers, and 14 096 nurses and midwives [33]. The upgrading and expansion of training institutions is ongoing. Schools of nursing doubled enrollment in 2011. For many years, Tanzania has trained assistant medical officers, a cadre between clinical officer and medical doctor; as the degree is not internationally recognized, their retention rate is high [34]. *Accessibility* The number of nurses and doctors per capita is low; nationally, it is increasing for both categories, but in 5 out of 25 regions, it was lower in 2015 than in 2014. Between 2010 and 2015, the number of new staff posted in public services was 77% of available positions (new employment permits approved) [35]. Recruitment in public services is made difficult by the competition from the not-for-profit private sector [36] and by emigration [37]. 74% of physicians work in urban areas, where their ratio to population is 17 times higher than in rural areas; 8% of health facilities are not functional because of the absence of personnel [33]. Absenteeism, low productivity [38–40]; difficulty in recruiting and retaining personnel, and management deficiencies [31] are considered as the main HRH problems. *Policy and regulation* To improve performance, the *Tanzania National eHealth Strategy 2013 – 2018* ^e^ proposes to give healthcare workers access to continuous professional development through e-learning and digital resources. Better remuneration of workers in the health sector is needed [39, 41], as are improved management practices and career development opportunities [42].

^b^ http://www.observatorisdmkindonesia.org/wp-content/uploads/2014/09/LIST-OF-RECOGNIZE-INSTITUTION-NURSING-EDUCATION-final.pdf

^c^There is a Sudanese Doctors’ Association of Ireland (http://www.sdui.org/)!

^d^This is the number of those traced by the Human Resources for Health Information System; the real number is estimated at 16 000

^e^ http://www.who.int/goe/policies/countries/tza_ehealth.pdf

## Case presentation

### Methods

#### Selection of country cases

The selection of countries was intentional, based on the following criteria for inclusion: to be from different WHO regions, to be of different economic level (see Table 1), and to have indicated progress in an informal monitoring of commitments conducted by WHO in mid-2014. The selection was limited to three countries because of resource constraints and because the study was defined as a pilot to inform a future research strategy for a broader follow-up.

#### Information search and sources

An initial web search was conducted in reference databases (PuBMed, Eldis) and web sites of WHO, World Bank, national ministries of health, and bilateral agencies (AusAID^3^, USAID) active in these countries to collect information from peer-reviewed articles, reports, and official documents in English, to help describe the situation of the health workforce in the country. Search terms were human resources/health workforce policies, data, and issues in the three selected countries for the period from 2000 to 2014. Most relevant information on topics relating to the commitments made in Recife was found in the gray literature. We did not expect to find published literature on the follow-up of the Recife commitments; the objective was to look for papers that helped understand the health workforce context in the three selected countries; in the web sites of WHO, the World Bank, AusAid, and USAID, we found country profiles, administrative documents, and project reports on the activities of these organizations in the three countries. The most useful sources were the web sites of national ministries and agencies of health; the main relevant policy documents were available in English. These are included in the list of references.

In addition to documents, information was collected in the field by five country-based co-authors with direct knowledge of HRH policy initiatives. One is a leader of the HRH Observatory in Sudan; two are WHO country staff, in Indonesia and Tanzania, whose mandate includes HRH; and two were high-ranking government officers in these latter two countries. A template for the collection of information was developed which covered actions explicitly taken to meet the Recife commitments, e.g., policy changes, management decisions, investments, actors involved in the design and implementation of these actions, results observed, facilitators/obstacles, and lessons learned. Data collection occurred between October 2014 and March 2015.

In this paper, we first describe the interventions which took place after the end of 2013 which can be linked to the Recife commitments. We then briefly discuss enabling and constraining factors that influenced commitment implementation, including their contents, actors involved, the policy processes, or the broader context.

### Results

While based on a common invite and global process linked to the Third Global Forum on HRH, the processes and contents of the commitments, as well as the monitoring and follow-up activities we conducted, were highly country-specific. The findings of the analysis are therefore presented separately by country.

#### Indonesia

Indonesia made two commitments: for each, five specific objectives were set, some with a timeline and others without (Table 3). Commitments were set at the national level; in addition, initiatives in line with the Recife commitments took place at the decentralized level, in particular with the support of AusAID. For instance, the Provincial Health Office in East Java promoted the adoption of a regulation (PERDA No. 7, 2014) to facilitate the recruitment of health personnel and to better define their roles and responsibilities, giving local governments the authority to deploy health workers on the basis of their own analysis of needs [4].

**Table 3:** Recife commitments, corresponding objectives, and progress reported: Indonesia

Recife commitments	Objectives	Progress reported
1: “To harmonize supply and demand of health workers in improving the quality of health workers”^f^	1.1: “to develop an annual HRH requirement plan as the reference/ consideration in processing the licensing of education institutions”	Accreditation of schools of medicine and dentistry started in 2009 by the National Board for accreditation of higher education institutions; it was extended to schools of pharmacy, nursing, midwifery, nutrition, and public health in 2011. The change introduced in 2014 was that the licensing of education institutions became the responsibility of an independent Accreditation Agency for Health Professional Education Institutions (LAM-PT.Kes)
	1.2: to develop an integrated HRH information system, using a HRH observatory approach as the reference by March 2014	A HRH Observatory has been established by the Ministry of Health in November 2014^g^ and training of its personnel started in January 2015; funding was provided by WHO and 10 professionals participated.
	1.3: to produce an annual HRH requirement plan by December 2013, and then every December	The upgrade of the HRH information system started in 2014, with funding from the Department of Foreign Affairs and Trade of Australia (DFAT). The Annual Planning for HRH for 2015 has been developed in collaboration with multiple stakeholders, including DFAT and WHO. This planning is based on the *HRH Plan 2011–2025*.
	1.4: to develop a distance learning program to upgrade the education level of nurses and midwives from Diploma 1 to Diploma 3 level in remote regions	Distance learning activities for nurses and midwifes have been conducted in two provinces: East Nusa Tenggara, with funding from Australia, and East Kalimantan, with funding from the regional government. The digitalization of training modules was initiated at the end of 2014.
	1.5: to develop a health workforce registration mechanism through competency certification (using exit exam as the certification exam) to ensure the competency of HW before registering to the health professional council	A national exit exam is in place for medicine and dentistry. XA similar exam was introduced for nurses and midwifes in 2014.^h^ It is planned to extend this mechanism to other health professions in 2016.
2: “To improve the HRH distribution and retention”	2.1 and 2. 2: affirmative action by provision of scholarships with bonding service to health workers in remote and underserved areas by December 2014 and to develop Guidelines of Scholarship with bonding service for remote underserved areas by June 2014	A program of scholarships for students accepting to work in remote regions was implemented in 2014.
	2.3: to recruit students from remote and underserved regions from November 2013 onwards	Recruitment of students from these regions has started in 2014.
	2.4 and 2. 5: to develop a task shifting model for health workers in remote areas by April 2014 and to develop Recommendations and Guidelines on task shifting by April 2014 and modules and curriculum of training by September 2014	Recommendations for task-shifting and training modules have been developed in 2014 as planned.

^f^We quote commitments and objectives verbatim. The information on follow-up was obtained directly from ministries of health by country correspondents

^g^ www.observatorisdmkindonesia.org/ Available only in Indonesian

^h^ http://www.observatorisdmkindonesia.org/wp-content/uploads/2015/01/3.-Indonesian-Nursing-Act-No.-38-year-2014.pdf)

The commitments were aligned to the Ministry of Health *Strategic Plan 2010–2014* and based on the *Indonesia Human Resources for Health Development Plan 2011–2025* (*HRH Plan*). The Recife commitments and objectives corresponded to proposals already made, in order to address deficiencies at the levels of availability, accessibility, and performance of health workers [4].

Representatives from government, professional associations, academia, health facilities, and international agencies participated in the formulation of the *HRH Plan* as members of a *Country Coordination and Facilitation* (CCF) Committee. Given the link of the commitments with the *HRH Plan*, the process of their adoption was a participative one which brought together numerous actors. Their implementation was planned in collaboration with national stakeholders and with development partners, mainly the WHO and AusAID, whose support accelerated the creation of a HRH Observatory and the strengthening of the HRH information system. Overall, there has been progress in implementing the actions in the commitments, with multiple interventions leading to complete or partial achievement of their objectives (Table 3). This was done in partnership with various health sector stakeholders in continuity with actions already planned to respond to the growing demands of the health system.

#### Sudan

The decision to make formal commitments at the Recife Forum was in continuity with previous policy interventions in the health sector, as the debate on the critical role of the health workforce was already going on.

The Federal Ministry of Health (FMOH) based its Recife commitments on the *National Human Resources for Health Strategic Plan 2012–2016*, focusing on improving the performance of health workers and on pre-service education. The main strategies were to strengthen management by increasing the number of managers with fit-for-purpose competencies and to expand the accreditation of education institutions and the upgrading of curricula (Table 4). These commitments fitted in the existing policy agenda and were therefore well accepted. Following the Recife commitments, HRH issues were pushed higher on the political agenda, as illustrated by the subsequent creation of a health workforce committee by the National Council for Health Care Coordination, chaired by the President of the Republic, which elevated health workforce issues as a whole-of-government issue rather than merely a health sector one.

**Table 4:** Recife commitments, corresponding objectives, and progress reported: Sudan

Recife commitments	Objectives	Progress reported
1: “To enhance performance”	1.1: “to improve the availability of adequate number of health managers, who have appropriate competencies and skills”	The Federal Ministry of Health (FMOH) mandated the Public Health Institute to implement diploma and master programs in public health, health services, hospital and disaster management, and human resources development; 400 candidates, mostly staff of state ministries of health, were enrolled in 2014. More than 300 obtained a diploma and reintegrated to their position at federal and state levels.
	1.2: “to enhance performance through the efficient critical management support systems - planning and budgeting; financial management; personnel management, infrastructure & logistics management; procurement and distribution of drugs and other commodities; information management and monitoring”	The FMOH invested in strengthening the planning, budgeting and monitoring, and information and personnel administration systems, but their full potential is yet to be realized, especially at decentralized levels. The FMOH and state ministries of health, with the support of the WHO and other partners, created a “planning platform” in 2014; it meets periodically and organizes training to build management capacity.
	1.3: to enhance performance through an enabling working environment: degree of autonomy, clear definition and communication of roles and responsibilities, fit between the roles and structures, existence of national standards, rules and procedures, regular meetings, and supportive supervision;	Despite efforts to develop standards, rules, and procedures, progress in improving the working environment is slow. In 2015, the Cabinet issued a directive on improving work environment and retention to address the health worker migration to Gulf States. This was based on recommendations of the Federal Ministry of Health.
	1.4: to enhance performance through updating the Continuing Professional Development (CPD) policy that in-service training/ continuous medical education is accredited as a means for licensing and relicensing;	In 2015, the Federal Ministry of Health, in collaboration with the Sudan Medical Council, initiated a policy process to develop guidelines for the accreditation of CPD and its linkages to licensing and promotion. A broad consultation is in process, and the adoption of the guidelines is planned to take place before mid-2016.
2: “To enhance quality of pre-service education”	2.1: “to enhance the quality of pre-service education through improved postgraduate and undergraduate curricula for medical, dental and pharmacist disciplines”	The FMOH, with support from WHO and the Sudan Medical Council, initiated a reform of medical education to align it with health service needs and strategies. A pilot curricular reform started in four medical schools; reform of dental and pharmacy curricula has yet to start. At the postgraduate level, the curricula of 20 postgraduate medical specialty programs were reviewed in 2014. The Sudan Medical Specialization Board (SMSB) is currently updating the curricula of other postgraduate programs. The SMSB is also leading a reform through a new strategy prepared in 2015 focusing on the expansion of training sites, decentralization of training management, introduction of nursing specialties, and strengthening accreditation. The SMSB increased added 10 new specialty programs, including nursing and midwifery.
	2.2: “To enhance quality of pre-service education through improved pre-service curricula for the allied medical and health professions”	The Academy of Health Sciences (AHS) is currently updating nursing, midwifery, and laboratory, medical, dental, and pharmacy assistants’ programs. In 2015, three new branches of the AHS were added at the locality level. In addition, over 800 community midwives went through a crash program and were deployed to underserved areas in 2015.
	2.3: “To enhance quality of pre-service education through the accreditation of postgraduate and undergraduate training facilities for medical, dental and pharmacist disciplines”	The Sudan Medical Council accreditation program, first established in 2008, has organized teams to conduct field visits to all medical schools in 2015. There are still no accreditation decisions, but the experience has triggered changes and facilitated resource mobilization for infrastructure and program development. The Council joined the World Federation of Medical Education “accrediting the accreditors” program; it has now applied to be accredited. There is no accreditation of dental and pharmacy schools, but standards for dental and pharmacy schools were reviewed and finalized in 2015 by the Sudan Medical Council. In 2015, the Sudan Medical Specialization Board developed standards for accrediting training sites and trainers. Application of these standards is underway [43].

After Recife, the Health Workforce Observatory and the FMOH HRH department received additional financial support to improve their infrastructure and technical capacity. This was facilitated by the signing of agreements in 2014 with GAVI and the GFATM which gave priority to health workforce strengthening. These agreements included measures to improve the quality of training and continuing professional development of health workers and of managers and to address the issues of attraction and retention of qualified personnel in regions with unmet needs [5, 6].

Medical education reform and the design of a continuing professional development (CPD) policy and of a process of recertification were supported by national institutions, including the Academy of Health Sciences, the Sudan Medical Council, and the Public Health Institute. External partners, e.g., WHO and the University of Leeds (England), provided technical assistance, particularly in the training of health managers.

Even though improvements in the health workforce situation are reported, major challenges remain: there is education room for further improvement in the quality of nursing, midwifery, and allied health and in the strengthening of accreditation mechanisms and of HRH management systems, and above all, the benefits of capacity-building efforts are eroded by the emigration of a significant number of graduates, especially medical specialists, managers, and technicians [7].

#### Tanzania

Tanzania’s Recife commitments were identified at a National Conference on “Health Workforce: Crucial to Meeting the Development Goals”^4^ in September 2013. More than 420 participants from government, civil society, faith-based and other private organizations, academia, and development partners participated and agreed to the adoption of three commitments (Table 5). The definition of the commitments was inspired by Malaysia’s “Big Fast Results” approach^5^ (called *Big Results Now* in Tanzania) which consists of identifying key areas where significant results can be obtained rapidly. This was applied to all sectors of the economy. In health, the process of identification of priorities and objectives started with a 6-week “Lab workshop” which involved 100 stakeholders from government, the private sector, civil society, and development partners. This led to the Recife commitments being well supported by the government and by stakeholders.

**Table 5:** Recife commitments, corresponding objectives, and progress reported: Tanzania

Recife commitments	Objectives	Progress reported
1: “To increase the availability of skilled health workers at all levels of health service delivery from 46 % to 64 % by 2017 based on staffing levels of 2013”	1.1: “To increase the density of health worker to population of the districts with below national average of 1.47 health workers per 1,000 population in 5 regions (Kigoma, Tabora, Rukwa, Shinyanga and Singida) from 0.73 health worker per 1,000 population to the national average”	During fiscal years 2013/2014 and 2014/2015, the 5 regions, which represent 18.5% of the total population, were allocated 20% of 19 566 new posts. Countrywide, the density of skilled health workers increased to 0.903 in 2014/15 after new posts were filled. In all 5 regions, the density has increased: Kigoma from 0.37 to 0.61, Tabora from 0.34 to 0.67, Rukwa from 0.54 to 0.70, Shinyanga from 0.57 to 0.62, and Singida went from 0.60 to 0.73, thus reaching the national average for 2013, but it remains below that of 2015. Out of 25 regions, 10 remained below the national average, 1 is “borderline,” and 14 are above [44]. There is also a proposal to legislate that students trained on public funds will not be registered until they have completed a compulsory 2-year period in rural areas.
	1.2: “To continue increasing production of skilled Health and Social workers from 4,364 in 2012 to 9,000 by 2017”	A *Production of Health Workers Plan (2014–2024)* has been approved; it outlines HRH objectives for the medium-term and provides a framework for short-term plan development. In 2014, the enrollment of allied health workers at certificate and diploma levels was 5 569, an increase of 77% respective to the previous year (3 143). For nurses and midwives, the increase was more modest (7.7%, from 5 135 to 5 533), and for doctors, pharmacists, dentists, and nursing officers, there was a decline from 1 890 to 1 810 [45].
	1.3: “To rationalize employment permits for health and social workers based on production and needs in all areas of technical professions”	The MoHSW developed a detailed 5-year recruitment plan which includes the expected production of health workers in each year [46].
2. “To increase financial base (Other Charges and Private sector investment) to operationalize the pay and incentive policy by 2017”	No specific objectives were specified	Tanzania has developed a plan to increase financial resources to attract and retain qualified health workers, and various measures are being taken: -A pay and incentive policy for public sector employees has been adopted, including subsistence, extra duty, risk, and on-call allowance increases; -Increase of opportunities for capacity building and professional development and establishment of distance learning centers; -Improvements in working environment at the level of accommodation, equipment, availability of medicines and supplies, and renovation and expansion of infrastructures; -Provision of basic amenities in rural areas: water, electricity, and transport; -Sensitization of students to apply to health training
2: “To develop and implement a Task Sharing Policy on HRH by 2017”	2.1: “To develop an operational guideline based on consolidated 2013 WHO guidelines on task sharing to enhance existing Production and Quality Assurance Systems by 2015”	A Task Sharing Policy Guideline [47] was endorsed by the MoHSW on 2 February 2016. These Policy Guidelines will scale up agreed task-sharing practices at all levels of the health care delivery system (dispensary, health center, and district hospital). The Guidelines cover the development of a regulatory framework, the provision of supervision, mentoring, follow-up at regular intervals, and the definition of roles and associated competencies.
	2.2: “To implement a system-wide approach that includes representation from other departments across different health cadres including professional associations, regulatory bodies, training institutions, accreditation bodies and policy makers to decide on common areas for task sharing across healthcare cadres by 2017”	The process of developing the Task Sharing Policy and Guidelines (see 2.1) was participatory. In September 2014, a stakeholder forum was convened, during which a research synthesis and evidence on task-sharing were presented, initial inputs on task-sharing were solicited, and practices and experiences with task-sharing were shared. Additional consultations involved professional councils, boards, and associations in 2015. The next step is to develop an implementation plan.

The commitments made by Tanzania include objectives targeting very specific density rates and targets for the number of health professional graduates to produce within a period of 3 years. However, the type of health workers these objectives refer to is not specified. The three commitments focus on improving the availability and accessibility of health workers in underserved regions, combining measures to increase the allocation of posts, even if modestly, to attract and retain new graduates, and to review the scopes of practice to allow workers with less training to perform tasks traditionally reserved to higher trained ones. Since November 2013, the implementation of the *HRH Strategic Plan 2014–2019* and of the *Production Plan for 2014–2024* has started. The *Health Sector Strategic Plan IV* (2015–2020) includes the Recife commitments [8]. The Ministry of Health and Social Welfare (MoHSW), in collaboration with line Ministries and with development partners, established a mechanism to monitor and report on these commitments and their related objectives, as part of the *HRH Strategic Plan 2014–2019*.

In spite of an average growth of 7% of its gross domestic product in the last 5 years, Tanzania’s health sector depends on external resources for close to 40% of total expenditure [9]. The lack of resources makes it difficult to increase the number of qualified health workers to the level proposed by national plans. Even though more health workers are educated, many end up emigrating or working in other sectors, which makes the achievement of the objectives announced in Recife difficult to attain.

### Discussion

The commitments made in Recife by representatives of Indonesia, Sudan, and Tanzania’s governments can be analyzed as policy statements. Reflecting on the work of various authors [10–13], we examine how policies are shaped by the interaction between their content, the processes of their adoption and implementation, the actors involved, and the context in which they take place. This is useful to structure the information collected, acknowledging that the short period of time covered and the volume and nature of the information do not permit broad generalizations.

#### Context

The three countries already had a HRH plan or equivalent document, from which their commitments were in fact derived. Indonesia had a *HRH Development Plan for 2011–2025*, Sudan one for 2012–2016, and Tanzania one for 2008–2013 and a subsequent one for 2014–2019, which incorporates the Recife commitments. The Recife Forum offered an opportunity, or in the words of Reich [11] a “political moment,” to give additional visibility to the HRH policy agenda in the three countries and to further engage policy-makers themselves and stakeholders who composed the delegations attending the international conference. In spite of a challenging political and economic environment, health workforce issues were already high on the three countries’ health policy agenda, which created a favorable environment to accept to make commitments in Recife and to initiate their implementation.

#### Content

In terms of content, the three countries’ commitments focused on the availability of health workers, on their geographical accessibility, on the quality of their education, and on strengthening governance, information systems, and management of the health workforce. This was seen as critical to improve the performance of the health service system. In Indonesia and Tanzania, a broad range of occupational groups were targeted. In Sudan, the focus has been mainly on physicians, which can be explained by the fact that the country is losing a significant proportion of its physicians to emigration. Sudan has also given specific attention to the availability of qualified managers and technicians. In Indonesia, the issue of accessibility to health workers is seen as the greatest challenge in view of the geography of the country and of the ethnic composition of the population. These are important challenges, but other complex issues, such as the extent and impact of dual practice or the review of scopes of practice, have not been included.

#### Process

The processes of adoption and of early implementation of the interventions proposed in the commitments varied from country to country. In Tanzania, a consultation of stakeholders was convened in order to reach a consensus on HRH policy objectives. The *Big Results Now* strategy used in this country was not specific to the health sector, where it was applied well after other sectors [14]. It aligned well with GHWA and WHO’s objective of encouraging countries to make commitments in Recife. In Sudan, the HRH Observatory already had the mandate to define health workforce policy objectives and was a sort of “policy entrepreneur” [11] which took the lead in formulating the commitments in consultation with representatives of stakeholders who are part of the Observatory. At the level of implementation, Tanzania and Sudan organized workshops and training activities to reinforce the capacities of managers and of government and stakeholder organizations’ technical staff to facilitate implementation. In Indonesia, the weak technical capacity remains a major challenge.

#### Actors

In the three countries, the Ministry of Health led the design and implementation of the commitments and their related objectives, but various national stakeholders, such as other ministries and government agencies, professional organizations, and education institutions, also participated actively. The support of external actors was determinant in every country: in Indonesia, a comprehensive partnership agreement with the Government of Australia, covering the period between 2011 and 2016, provided the resources and technical support to implement the Recife commitments. Sudan has benefited from the technical support of the University of Leeds; in Tanzania, USAID-funded technical assistance by the non-governmental organizations IntraHealth International and its Capacity Plus program, and by Management Sciences for Health, supported the process of health workforce development. In the three countries, WHO played an active role in bringing and keeping health workforce issues on the policy agenda and in supporting policy and decision-makers in the formulation of their HRH strategies.

### Limitations

The study has limitations: first, at this stage, quantitative data are limited in most cases and progress in implementing the commitments can only be assessed primarily on the basis of qualitative information, such as policy decisions, establishment of governance mechanisms, or statements of policy-makers. Second, as in all case studies, the external validity of the findings remains weak. Third, the selection criteria introduced a bias by targeting only countries reporting “progress” in implementing their Recife commitments in the informal follow-up of mid-2014; therefore, the findings should not be interpreted as representative of the situation in the broader group of 57 countries that made commitments but rather as illustrative of the potential of the commitment process to contribute to advancing the HRH agenda. There would be an obvious benefit in looking at the experience of countries which have not been able to make progress as planned in order to identify the barriers they have encountered. There is also a potential bias due to the proximity of some of the co-authors with government decision-makers; these may have wanted the findings to reflect well on what their country has done to achieve Recife commitments. This was addressed by triangulating findings from different information sources and validating them with documentary evidence. The involvement of WHO staff in the analysis was sought also to provide an independent perspective that could validate the information provided by other stakeholders. Finally, we assessed interventions at the national level, whereas there were also measures at the decentralized level in the three countries. For example, in Indonesia, provincial governments have the responsibility of managing their health workforce and many have taken initiatives such as opening education institutions adapted to their specific needs and creating their own stakeholder platforms in support of health workforce development [15].

In order to go beyond the descriptive approach presented here, further research will be required to answer questions such as the following: to what extent the various categories of stakeholders contributed to following up on the commitments? For instance, what has been the influence of external actors? Did the actions identified in this review produce the expected effects? Depending on the answer, what were the factors (relating to context, actors, processes) that explain success or failure? This type of deeper analysis will be possible when more time has passed and adding field work to document analysis.

## Conclusions

At the global level, most countries experience problems of health worker availability, accessibility, and performance; these typically include an insufficient number of qualified health workers, imbalances in their skill mix, in their distribution by levels of service and by geographical regions, the lack of alignment between education content and processes and service needs, or a weak regulation of private practice. However, there is no blueprint for policy changes which countries can rely on to address these deficiencies. Each country’s historical, economic, and political context and health needs are specific, as illustrated by the three country examples, and therefore, policy options need to be adapted accordingly. Countries can learn from the experience of others, but in the end, they have to design their own HRH strategies.

The factors that lead to the success or failure of HRH initiatives may vary, but the existence of a strong and continuous commitment of decision-makers has been recognized to be a critical enabler of effective action anywhere [1]. Our review suggests that the commitment process had some success in creating a window of opportunity for accelerated action on health workforce development, as evidenced by progress in implementation of several follow-up actions and by the subsequent inclusion of these commitments in national strategic and monitoring frameworks. The existence of policy documents that explicitly recognize and document HRH problems and that set out explicit relevant policy goals and targets, the involvement of the main stakeholders, and the availability of external support were the main facilitating factors that enabled countries to engage in the commitment process and to use it in addressing known HRH challenges. These findings are consistent with the broader evidence on HRH policy change which has repeatedly underscored the importance of including explicit HRH policy objectives in national plans and strategies and of broadening the participation of different sectors and constituencies and aligning their support with the national agenda [1, 16, 17].

The example of Sudan also suggests that the existence of a dynamic coordination mechanism, such as an HRH Observatory, can facilitate the whole process by creating a platform to bring together the main actors involved in health workforce development at the national level and providing technical support to the design and implementation of HRH interventions. The commitment process showed clearly that lack of awareness or ambition on the part of national governments or their international partners is not the problem. Country strategies—and the HRH commitments which were largely based on them—recognized the magnitude of the challenges and proposed ambitious responses. The main challenges which countries face are to mobilize political will and maintain the support of decision-makers, as their leadership is essential in the following: ensuring effective intersectoral governance and collaboration; protecting public interest from undue influence of special interests; relaxing restrictive public sector and civil service policies when these prevent providing health workers with adequate incentives and motivation; enabling the emergence of technical excellence by adopting and reinforcing meritocratic selection criteria for senior positions in the public health sector administration; and in mobilizing financial resources for the health workforce investment agenda, aligning education, finance, labor, and health policies [18].

## Footnotes

1 The Forum took place in Recife (Brazil) on 10–13 November 2013; http://www.who.int/workforcealliance/forum/2013/en/.

2 A template for the formulation of measurable and dated objectives was sent to participants prior to the Forum [48]. The full list is available at http://www.who.int/workforcealliance/forum/2013/hrh_commitments/en/index.html.

3 Now changed to Department for Foreign Affairs and Trade (DFAT) of Australia.

4 One of the stated objectives of the Conference was “To propose and advocate for national commitments to be declared by the Tanzania delegation at the 3rd Global Forum for HRH in November 2013”; http://pdf.usaid.gov/pdf_docs/PA00JZHP.pdf.

5 http://www.pemandu.gov.my/gtp/Big_Fast_Result-@-Big_Fast_Results.aspx.
